# Shared Decision-Making With a Surrogate for Life-Sustaining Treatment of Critically Ill Patients: Protocol for a Scoping Review

**DOI:** 10.2196/83284

**Published:** 2026-01-21

**Authors:** Yoshiyasu Ito, Mika Moriyama, Akemi Nasu, Keiko Kamitani, Shimpei Hayashi, Satoko Ono, Yasuko Sumida, Keiko Matsumoto, Misae Ito

**Affiliations:** 1 Faculty of Nursing Science, Tsuruga Nursing University, 2-1, Kizaki 78, Tsuruga City, Fukui, 914-0814, Japan, 81 0770-20-5519; 2 Faculty of Nursing, Kobe City College of Nursing, Kobe, Japan; 3 Department of Nursing, Sanyo Gakuen University, Okayama, Japan; 4 Graduate School of Medicine, Yamaguchi University, Ube, Japan; 5 Graduate School of Medicine, Kagawa University, Miki, Japan

**Keywords:** intensive care unit, ICU, life-sustaining treatment, protocol, scoping review, shared decision-making, Preferred Reporting Items for Systematic Reviews and Meta-Analyses, PRISMA

## Abstract

**Background:**

Shared decision-making (SDM) is a collaborative process that integrates patients’ values and preferences into health care decisions. In intensive care units, patients who are critically ill often lack the capacity to make decisions, necessitating surrogates to make complex choices regarding life-sustaining treatments (LSTs).

**Objective:**

This scoping review aims to assess the range of research conducted on surrogate SDM for LSTs among patients who are critically ill over the past decade and highlight areas where current research remains limited.

**Methods:**

This scoping review will follow the Joanna Briggs Institute methodology and adhere to the PRISMA-ScR (Preferred Reporting Items for Systematic Reviews and Meta-Analyses Extension for Scoping Reviews) reporting guidelines. Studies will be included if they examine SDM involving surrogates of adult patients who are critically ill in relation to LST decisions within intensive care unit settings. SDM is defined using 4 criteria: participation of both health care professionals and surrogates, mutual information sharing, consensus building, and agreement on treatment based on the patient’s values and preferences. A comprehensive search will be performed across PubMed, CINAHL, PsycInfo, CENTRAL, and Ichushi-Web for English- and Japanese-language studies published between 2016 and 2025. Eligible study designs will include quantitative, qualitative, and mixed methods research. Title and abstract screening, as well as full-text selection, will be conducted independently by 2 reviewers using Rayyan. Data will be extracted on study characteristics, SDM definitions, participant roles, and key findings. Results will be synthesized descriptively and presented in tables and narrative summaries to identify research gaps and inform future investigations.

**Results:**

As of June 13, 2025, the literature search has been completed. A total of 2899 citations were identified through the specified database searches, and 527 (18.2%) duplicates were removed. Title and abstract screening are currently in progress, and full-text review is expected to be completed by September 2025.

**Conclusions:**

This scoping review will systematically map recent evidence on surrogate SDM in the context of LST decisions for patients who are critically ill. By synthesizing diverse studies, it will identify challenges faced by surrogates and summarize existing interventions that aim to improve SDM processes. The findings are expected to inform future interventions and policies and advance patient- and family-centered care in critical care settings.

## Introduction

### Background

In intensive care units (ICUs), patients frequently lack the capacity to make decisions for themselves, and therefore, decisions regarding their health care are delegated to surrogates. To achieve patient-centered care, shared decision-making (SDM) remains essential even when surrogate decision-makers are involved. SDM has been described as a clinician-focused approach that facilitates the provision of family-centered care [1] and is recommended as the primary model for decision-making in the ICU [2]. SDM in the ICU is defined as follows: “SDM is a collaborative process that allows patients—or their surrogates—and clinicians to make healthcare decisions together, taking into account the best available scientific evidence as well as the patient’s values, goals, and preferences” [2].

The process of surrogate decision-making in the ICU is frequently reported to be a highly challenging experience [3], with approximately 19% of ICU patient surrogates experiencing moderate to severe long-term decisional regret [4]. Furthermore, such negative experiences with surrogate decision-making have been shown to increase the long-term risk of psychological impairment among surrogates [5,6]. SDM has been shown to improve patient satisfaction and the quality of care [7], and it is also expected to alleviate the negative experiences associated with surrogate decision-making in the ICU, serving as a crucial component in supporting surrogates [1].

SDM is indicated when patients or their surrogates struggle to identify achievable goals or when a patient’s clinical trajectory changes substantially as such treatment decisions warrant the use of SDM [8]. Patients in ICUs are critically ill, and situations involving multiple rational options are rare; however, in decisions regarding life-sustaining treatments (LSTs), SDM remains applicable because choices must be made among several possible interventions. Surrogates of patients who are critically ill are often required to make difficult decisions about LSTs, such as whether to withhold, withdraw, or escalate life-sustaining interventions. Decision-making regarding LSTs is highly complex and challenging as it requires balancing the patient’s best interests, the surrogate’s values, and their understanding of the patient’s preferences. Consequently, surrogates frequently experience strong feelings of regret concerning their decisions [4].

In the ICU, where medical decisions often carry significant ethical and emotional implications, SDM serves as a critical framework for aligning medical care with patients’ wishes. It is also a key approach to reducing decisional conflict among surrogates and guiding them through the process of making difficult choices [9,10]. More recently, a consensus framework has been developed to guide decision-making processes regarding the continuation or limitation of LSTs in ICU patients, and SDM has been increasingly recommended as an essential component of such decision-making [11].

Building on this growing emphasis on SDM in the ICU, previous systematic reviews have summarized evidence related to SDM in decisions about LSTs in the ICU [12-14]. However, these reviews primarily include studies conducted before a standardized definition of SDM in the ICU was established. For many years, the conceptualization of SDM remained inconsistent [15,16]. In recent years, research on SDM has progressed rapidly [17], and within the ICU context, the clarification of its definition and its wider adoption in both scholarly work and clinical practice did not occur until 2016 [2]. Although several studies examining SDM in decisions about LSTs in the ICU have been published in recent years [18-20], no recent scoping review has systematically assessed the current range of research or identified existing knowledge gaps.

### Review Objectives

The aim of this scoping review is to clarify the extent and nature of the evidence on SDM involving surrogates in LST decisions for patients who are critically ill. This review seeks to provide valuable insights into the current state of SDM in critical care, identify existing knowledge gaps, and suggest directions for future research.

### Review Questions

The research questions for this scoping review are as follows:

1. What studies have examined SDM involving surrogates in LST decisions for patients who are critically ill, and what are their key findings?
2. What types of SDM-related interventions have been implemented by health care professionals to support surrogate decision-making regarding LSTs for patients who are critically ill?

## Methods

### Study Design

This review aims to clarify the extent and nature of the evidence on SDM involving surrogates in LST decisions for patients who are critically ill. It does not aim to synthesize evidence for clinical decision-making or generate practice recommendations; rather, its primary objective is to map the existing body of evidence. Scoping reviews are an appropriate methodology for such purposes as they are designed to determine the scope and coverage of research on a given topic and provide a clear understanding of the volume, focus, and characteristics of the available evidence [21]. Therefore, a scoping review approach was considered the most suitable methodology for this study. The proposed scoping review will be conducted in accordance with the Joanna Briggs Institute methodology for scoping reviews [22], and it will be reported following the PRISMA-ScR (Preferred Reporting Items for Systematic Reviews and Meta-Analyses Extension for Scoping Reviews) guidelines (Checklist 1) [23]. This review protocol has been registered on the Open Science Framework [24].

### Inclusion Criteria

#### Population

This scoping review will include studies on SDM involving surrogates who participate in treatment and care decision-making for adult patients aged ≥18 years. Surrogates include not only family members such as spouses, children, or parents but also individuals legally designated to make health care decisions on the patient’s behalf, including legal guardians and those holding durable power of attorney for health care.

#### Concept

This scoping review will include studies on SDM between health care professionals and surrogates of patients who are critically ill and lack decision-making capacity due to disease progression or functional impairment, including cognitive dysfunction, delirium, decreased level of consciousness, or other neurological impairments.

SDM for patients who are critically ill is defined as a collaborative process that enables patients—or their surrogates—and clinicians to make health care decisions together, taking into account the best available scientific evidence and the patient’s values, goals, and preferences [2]. According to Charles et al [25], SDM is characterized by four core features: (1) at least 2 participants—the health care professional and the patient—are involved, (2) both parties share information, (3) both parties engage in building a consensus about the preferred treatment, and (4) an agreement is reached on the treatment to be implemented. Accordingly, in this scoping review, SDM will be defined as meeting all the following criteria: (1) at least 2 participants—the patient’s surrogate and the health care professional—are involved; (2) both parties share information; (3) both parties take steps to build a consensus on the preferred treatment based on the patient’s values, goals, and preferences; and (4) an agreement is reached on the treatment to be implemented.

Studies involving decision-making support interventions—such as decision coaching, decision aids, and question prompt sheets—will be included if these constitute part of the SDM process and meet the 4 criteria described above. Studies that do not fulfill the SDM definition criteria or that focus on SDM involving patients with decision-making capacity will be excluded.

#### Context

This scoping review will include studies examining the decision-making process surrounding LSTs for patients who are critically ill. LST does not have a universally accepted definition across countries or clinical guidelines. Comparative analyses indicate that the scope of LST varies substantially by jurisdiction and often includes interventions aimed at maintaining vital organ function or preventing imminent death [26]. International consensus statements similarly define LSTs as treatments such as invasive mechanical ventilation, vasopressor or inotrope support, renal replacement therapy, extracorporeal life support (extracorporeal membrane oxygenation), cardiopulmonary resuscitation, and artificial nutrition or hydration [27-29]. Given this heterogeneity, this scoping review will include studies that explicitly refer to treatment decisions as “life-sustaining treatment,” and we will extract and map each study’s operational definition of LST.

For the purpose of this review, LST-related decision-making encompasses not only decisions regarding the initiation of such treatments but also decisions to withhold or withdraw them. These decisions most commonly occur in ICUs, where patients are critically ill and often require advanced medical interventions to sustain life. The ICU context is particularly significant because the time available for decision-making is often limited and the decisions made may have profound implications for patient survival. As such, these decisions are highly complex and ethically challenging, involving judgments about whether to continue or discontinue interventions that directly affect life or death. Studies addressing treatments or care other than LSTs, those conducted in settings outside the ICU, or those spanning multiple clinical environments (eg, ICUs and surgical wards) will be excluded.

### Types of Sources

This scoping review will consider both experimental and quasi-experimental study designs, including randomized controlled trials, nonrandomized controlled trials, before-and-after studies, and interrupted time-series studies. In addition, analytical observational studies—including prospective and retrospective cohort studies, case-control studies, and analytical cross-sectional studies—will be considered for inclusion. This review will also include descriptive observational study designs, such as case series, individual case reports, and descriptive cross-sectional studies. Studies focusing on qualitative data will also be considered, including but not limited to designs such as phenomenology, grounded theory, ethnography, qualitative description, action research, and feminist research. Although review articles will be excluded from the final analysis, the reference lists of all relevant review articles will be examined to identify additional eligible primary studies. Review articles, protocol papers, opinion papers, letters, editorials, commentaries, and gray literature (eg, conference proceedings, government documents, and institutional websites) will be excluded.

### Search Strategy

Although only the literature search has been completed to date, a 3-step search strategy was used. An initial limited search of PubMed was conducted to explore the range of terminology used in the literature and identify key articles relevant to SDM in ICUs. During this preliminary search, the titles and abstracts of retrieved articles were reviewed to extract frequently used text words and indexing terms (MeSH [Medical Subject Headings] terms). These terms were analyzed and incorporated into the development of the final comprehensive search strategy. The text words contained in the titles and abstracts of the relevant articles, as well as the indexing terms used to describe those articles, were used to construct the full search strategy (Multimedia Appendix 1). Specific keywords and indexing terms from each database and information source were integrated into the overall search strategy. The databases searched included PubMed, CINAHL Ultimate (EBSCO), PsycInfo (EBSCO), CENTRAL, and Ichushi-Web (Japan Medical Abstracts Society). Studies published in English or Japanese between 2016 and 2025 were included. The database searches were completed on June 13, 2025.

### Study Selection

Following the database searches, all identified citations will be collated and uploaded to the Rayyan software (Qatar Computing Research Institute), and duplicates will be removed. After a pilot screening, titles and abstracts will be reviewed by 2 independent reviewers to assess eligibility according to the inclusion criteria. Potentially relevant sources will be retrieved in full, and their citation details will be imported into Rayyan. Two independent reviewers will then assess the full texts of the selected citations in detail against the inclusion criteria. Reasons for excluding sources that do not meet the inclusion criteria will be documented and reported. Any disagreements between reviewers at any stage of the selection process will be resolved through discussion or, if necessary, consultation with an additional reviewer. The results of the search and study selection process will be presented using the PRISMA (Preferred Reporting Items for Systematic Reviews and Meta-Analyses) 2020 flow diagram [30]. The study selection process is scheduled to take place between June 2025 and August 2025.

### Data Extraction

Data will be extracted using a standardized data extraction tool developed by the reviewers (the tool will be cited or appended). Extracted data will include the following: source details (eg, citation information, country, and study design), study purpose, definition of LST and SDM, types of health care professionals involved in SDM, context or setting, participants, methodology, and main findings. A draft data extraction form is provided in Multimedia Appendix 2. The extraction tool will be refined and updated as necessary during data extraction from each included source, and all modifications will be documented in the final scoping review. Any disagreements arising during data extraction will be resolved through discussion or consultation with an additional reviewer. Data extraction is scheduled to be conducted between September 2025 and October 2025 following the completion of the study selection process.

### Data Analysis and Presentation

In this review, data will be primarily presented in tabular form; however, other formats that better illustrate the results will also be considered. A detailed mapping will be performed by describing each study within relevant domains, including country, study design, purpose, setting, participants, methodology, and key findings. When more than 2 studies address a single domain, the findings will be summarized narratively, and the evidence will be mapped descriptively. Data analysis and presentation are scheduled to be conducted between November 2025 and December 2025 following the completion of data extraction. The results of this scoping review are expected to be published from January 2026 onward.

## Results

As of June 13, 2025, the literature search has been completed. A total of 2899 citations were identified through the specified database searches, and 527 (18.2%) duplicates were removed. Title and abstract screening (conducted by YI, MM, AN, KK, SH, SO, YS, KM, and MI) is currently in progress, and full-text review is expected to be completed by September 2025.

## Discussion

### Anticipated Findings

In ICUs, patients who are critically ill frequently lose the capacity to make health care decisions, and responsibility for these decisions often falls to surrogate decision-makers [2,31]. Although SDM has been endorsed as the preferred framework for aligning medical treatment with patient values, its application in surrogate decision-making for LSTs remains limited [32]. Previous research has underscored the complexity of surrogate decision-making, including decisional conflict, regret, and psychological burden [3,33-35]. However, evidence on how SDM processes specifically address or mitigate these challenges in the ICU remains fragmented. To our knowledge, this scoping review will be the first to systematically map the extent and nature of the evidence on SDM involving surrogates in LST decisions for patients who are critically ill.

This review will clarify how SDM has been conceptualized and operationalized in the ICU setting, identify the types of health care professional interventions that have been implemented, and delineate the roles of these professionals within SDM processes. In ICUs, SDM has been implemented as an interprofessional process that requires collaboration among physicians, nurses, and other health care professionals [36,37]. This review will further contribute to advancing understanding of SDM by clarifying the specific functions of each professional group within these collaborative processes. Moreover, by synthesizing diverse study designs and methodologies, this review will provide a comprehensive overview of the current state of knowledge and identify areas in which empirical evidence remains insufficient. The findings are expected to inform the development of future interventions aimed at enhancing surrogate support and strengthening the integration of SDM into critical care practice.

The results of this scoping review are expected to contribute to both clinical practice and research in several ways. First, by mapping existing interventions and the roles of health care professionals in surrogate SDM, this review will provide valuable insights into multidisciplinary approaches that may help reduce decisional burden. Second, it will help identify which components of SDM are most effective in improving surrogate experiences, such as structured communication tools, decision aids, and family conferences.

A major strength of this review is the use of the Joanna Briggs Institute methodology and adherence to the PRISMA-ScR reporting guidelines [22,23], which ensure a systematic and transparent approach to evidence mapping. The inclusion of a wide range of study designs encompassing both quantitative and qualitative research will facilitate a comprehensive understanding of the topic. Furthermore, by incorporating literature published in both English and Japanese, this review may capture cultural and contextual variations that enrich the interpretation of the findings.

However, several limitations should be acknowledged. Restricting the search to publications from 2016 onward may exclude earlier yet potentially relevant studies on SDM. Similarly, limiting the review to English- and Japanese-language publications may result in the omission of findings from other cultural contexts where SDM practices differ. Another limitation is that, as a scoping review, this study will not involve an assessment of the methodological quality or risk of bias of the included studies, which may limit the strength of conclusions regarding intervention effectiveness. Despite these limitations, this review will represent an important initial step toward consolidating the existing evidence and identifying key gaps to guide future systematic reviews and interventional research.

### Conclusions

Overall, this scoping review will systematically map recent evidence on surrogate SDM in the context of LST decisions for patients who are critically ill. By synthesizing diverse studies, it will identify the challenges faced by surrogates and summarize existing interventions that aim to improve SDM processes. The knowledge generated will not only inform the development of future interventions and policies but also advance patient- and family-centered care in critical care settings.

## Supplementary material

10.2196/83284 Multimedia Appendix 1 Preliminary search strategy for PubMed.

10.2196/83284 Multimedia Appendix 2 Data extraction instrument.

10.2196/83284 Checklist 1 PRISMA-ScR checklist.

## Abbreviations

ICU: intensive care unit
LST: life-sustaining treatment
MeSH: Medical Subject Headings
PRISMA: Preferred Reporting Items for Systematic Reviews and Meta-Analyses
PRISMA-ScR: Preferred Reporting Items for Systematic Reviews and Meta-Analyses Extension for Scoping Reviews
SDM: shared decision-making
