# Supplementary material for: Shared Decision-Making With a Surrogate for Life-Sustaining Treatment of Critically Ill Patients: Protocol for a Scoping Review
Source: JMIR Res Protoc. 2026 Jan 21;15:e83284. doi: 10.2196/83284 (PMC12822866; doi:10.2196/83284)
Supplement: Multimedia Appendix 1 [file resprot-v15-e83284-s001.docx]

**S1 Table. Preliminary search strategy for the PubMed**

Preliminary search conducted in PubMed: March 26, 2025

| **Search** | **Query** | **Records retrieved** |
| --- | --- | --- |
| #1 | ((((((((((((((((((((((((((((("Family"[Mesh]) OR (famil*[Title/Abstract])) OR (surrogate*[Title/Abstract])) OR ("Caregivers"[Mesh])) OR (caregiver*[Title/Abstract])) OR (carer*[Title/Abstract])) OR (spous*[Title/Abstract])) OR (husband*[Title/Abstract])) OR (wife*[Title/Abstract])) OR (partner*[Title/Abstract])) OR (parent*[Title/Abstract])) OR (mother*[Title/Abstract])) OR (father*[Title/Abstract])) OR (son[Title/Abstract])) OR (sons[Title/Abstract])) OR (daughter*[Title/Abstract])) OR (sibling*[Title/Abstract])) OR (brother*[Title/Abstract])) OR (sister*[Title/Abstract])) OR (grandparent*[Title/Abstract])) OR (grandfather*[Title/Abstract])) OR (grandmother*[Title/Abstract])) OR (relative*[Title/Abstract])) OR (bereaved[Title/Abstract])) OR (loved one*[Title/Abstract])) OR (kinship*[Title/Abstract])) OR (next of kin[Title/Abstract])) OR (kindred*[Title/Abstract])) OR (significant other*[Title/Abstract])) OR (legal guardian*[Title/Abstract]) | 4,216,995 |
| #2 | (((((((((((("Decision Making, Shared"[Mesh]) OR (Shared Decision-Making[Title/Abstract])) OR (Participatory Decision Making[Title/Abstract])) OR (Patient Participation[Title/Abstract])) OR (Patient Involvement[Title/Abstract])) OR (Patient Activation[Title/Abstract])) OR (Patient Engagement[Title/Abstract])) OR ("Patient Participation"[Mesh])) OR ("Decision Making"[Mesh])) OR (decision Mak*[Title/Abstract])) OR (surrogate decision making[Title/Abstract])) OR (surrogate decision-making[Title/Abstract])) OR (SDM[Title/Abstract]) | 476,195 |
| #3 | ((((((((((((((((((("Life Support Care"[Mesh]) OR ("Resuscitation"[Mesh])) OR (life-sustaining treatment*[Title/Abstract])) OR (life-prolonging treatment*[Title/Abstract])) OR (life support care[Title/Abstract])) OR (treatment escalation[Title/Abstract])) OR (ceiling of treatment[Title/Abstract])) OR (ceiling of care[Title/Abstract])) OR (treatment limitation[Title/Abstract])) OR (withdraw*[Title/Abstract])) OR (withhold*[Title/Abstract])) OR ("Terminally Ill"[Mesh])) OR ("Terminal Care"[Mesh])) OR ("Palliative Care"[Mesh])) OR ("Hospice and Palliative Care Nursing"[Mesh])) OR (terminal care[Title/Abstract])) OR (palliative care[Title/Abstract])) OR ("Palliative Medicine"[Mesh])) OR (palliative medicine[Title/Abstract])) OR (end of life care[Title/Abstract]) | 406,647 |
| #4 | ((((((("Intensive Care Units"[Mesh]) OR ("Critical Care Nursing"[Mesh])) OR ("Critical Illness"[Mesh])) OR ("Critical Care"[Mesh])) OR (ICU[Title/Abstract])) OR (intensive care unit*[Title/Abstract])) OR (critically ill*[Title/Abstract])) OR (intensive care[Title/Abstract]) | 353,939 |
| #5 | #1 AND #2 AND #3 AND #4 | 2,046 |
| #6 | #4 Filters: from 2016 - 2025 | 827 |
