# Supplementary material for: Shared Decision-Making With a Surrogate for Life-Sustaining Treatment of Critically Ill Patients: Protocol for a Scoping Review
Source: JMIR Res Protoc. 2026 Jan 21;15:e83284. doi: 10.2196/83284 (PMC12822866; doi:10.2196/83284)
Supplement: Multimedia Appendix 2 [file resprot-v15-e83284-s002.docx]

**S2 Table. Data extraction instrument**

| **Evidence source Details and Characteristics** | |
| --- | --- |
| Citation details | |
| First author, year |  |
| date |  |
| title |  |
| doi |  |
| Country |  |
| Study Design |  |
| Purpose |  |
| Definition of LST |  |
| Definition of SDM |  |
| Types of healthcare professionals participating in SDM |  |
| Context/Setttings |  |
| Participant (e.g., number, age(mean), sex (number, %), disease) *If an intervention is present, describe both the intervention group and the control group separately.) |  |
| Methodology  *If an intervention is present, describe the details of the intervention and the outcomes.) |  |
| Main Results |  |
